# Interaction effects of multimorbidity and frailty on adverse health outcomes in elderly hospitalised patients

**DOI:** 10.1038/s41598-022-18346-x

**Published:** 2022-08-19

**Authors:** Sanja Lujic, Deborah A. Randall, Judy M. Simpson, Michael O. Falster, Louisa R. Jorm

**Affiliations:** 1 Centre for Big Data Research in Health, University of New South Wales, Sydney, Australia; 2 Women and Babies Research, The University of Sydney Northern Clinical School, Sydney, Australia; 3 School of Public Health, The University of Sydney, Sydney, Australia

**Keywords:** Risk factors, Health services, Epidemiology

## Abstract

We quantified the interaction of multimorbidity and frailty and their impact on adverse health outcomes in the hospital setting. Using aretrospective cohort study of persons aged ≥ 75 years, admitted to hospital during 2010–2012 in New South Wales, Australia, and linked with mortality data, we constructed multimorbidity, frailty risk and outcomes: prolonged length of stay (LOS), 30-day mortality and 30-day unplanned readmissions. Relative risks (RR) of outcomes were obtained using Poisson models with random intercept for hospital. Among 257,535 elderly inpatients, 33.6% had multimorbidity and elevated frailty risk, 14.7% had multimorbidity only, 19.9% had elevated frailty risk only and 31.8% had neither. Additive interactions were present for all outcomes, with a further multiplicative interaction for mortality and LOS. Mortality risk was 4.2 (95% CI 4.1–4.4), prolonged LOS 3.3 (95% CI 3.3–3.4) and readmission 1.8 (95% CI 1.7–1.9) times higher in patients with both factors present compared with patients with neither. In conclusion, multimorbidity and frailty coexist in older hospitalized patients and interact to increase the risk of adverse outcomes beyond the sum of their individual effects. Their joint effect should be considered in health outcomes research and when administering hospital resources.

## Introduction

Frailty and multimorbidity are gaining attention with the noted global increase in the average age of populations. Frailty denotes a state of increased vulnerability of individuals to stressors from the accumulated consequences of morbidities or their treatments^1^ and multimorbidity is a “co-occurrence of two or more chronic conditions where one is not necessarily more central than the others”^2^. Both states have been associated with higher healthcare utilisation, costs and mortality,^3–6^ and are used by health policy planners to identify both high-need patients and those at risk of adverse health events. However, while patients experiencing frailty and multimorbidity often require additional care, the specific care needs and prognosis may vary between them^7^.

Currently there is no consensus on standardised measurement of either multimorbidity or frailty, with choice of the measure often driven by data availability and the study setting. Routinely collected data are increasingly being used in ageing health research^8^, giving opportunities to define frail and multimorbid individuals from their full health care history. A range of claims-based frailty indices were developed recently^3^, with one such measure using administrative data for hospitalised patients being the Hospital Frailty Risk Score (HFRS)^9^. HFRS helps identification of patients with characteristics of frailty, and at risk of adverse healthcare outcomes including mortality, prolonged hospital stays and unplanned readmissions. The score has been validated in medical^10,11^ and surgical^12,13^ patients.

While various definitions of frailty and multimorbidity exist, they are intrinsically linked with each other, with most frail patients being multimorbid, and one-sixth of multimorbid adults presenting with frailty^14^. The National Institute for Health and Care Excellence (NICE) guidelines recommends frailty to be considered when managing older adults with multimorbidity^15^. Since many patients experience both multimorbidity and frailty, there is potential that they interact to increase risk of adverse outcome. For example, multimorbid patients who are in a frail state may experience higher risk of complications, or be predisposed to more severe and ongoing complications, than multimorbid patients with low frailty. Investigating how an additional health state impacts on the health outcomes in patients already experiencing another is of importance as it can help identify those at greater risk and in need of further targeted health services.

Interactions between multimorbidity and frailty can be measured on different scales. Interaction on an additive scale would imply that the combined effect of multimorbidity and frailty is greater than the sum of their individual effects, whereas interaction on a multiplicative scale would imply that their combined effect is greater than the product of their individual effects. While interactions on an additive scale are more important in public health^16,17^ due to the ease of interpretation of absolute rather than relative numbers of patients who might benefit from an intervention, quantifying the magnitude and scale of the interaction is crucial for understanding the extent to which the presence of one condition amplifies the effect of the other. Given there has been a rapid increase in the prevalence of both multimorbidity and frailty in patient populations^18,19^, identifying such amplifying factors is critical to inform future health care resource planning.

To our knowledge, no studies to date have examined interaction between the two factors in hospital patients, who are at higher risk of complications and adverse outcomes. The purpose of our study was to quantify the prevalence of multimorbidity and frailty, and their association with outcomes, in a large observational cohort of elderly Australian hospital patients. Specifically, we aimed to (1) estimate the joint effect of HFRS and multimorbidity with hospital outcomes (30-day mortality, 30-day readmission, prolonged length of stay), and (2) characterise the type of interaction between multimorbidity and frailty.

## Results

### Study cohort

Our cohort included a total of 257,535 patients aged 75 and over with an unplanned hospital admission during 2010–2012. The majority of the admissions (86%) were medical in nature, with a smaller proportion (9.1%) being for surgery, and the remainder in the other category. The median patient age was 83.3 years (interquartile range 79.2–87.7) and most patients were female (57.2%). Other cohort characteristics are shown in Table 1.

**Table 1:** Cohort description at the time of index hospitalisation 2010–2012, by multimorbidity and frailty risk.

	Total	Multimorbidity and frailty risk				*p*-value
		Neither	Elevated frailty risk only	Multimorbid only	Both	
	N = 257,535	n = 81,788	n = 51,279	n = 37,949	n = 86,519	
**Sex, n (%)**						
Male	110,125 (42.8)	34,363 (42.0)	17,134 (33.4)	19,587 (51.6)	39,041 (45.1)	< 0.01
Female	147,410 (57.2)	47,425 (58.0)	34,145 (66.6)	18,362 (48.4)	47,478 (54.9)	
Median age (IQR)	83.3 (79.2–87.7)	81.9 (78.3–86.2)	84.9 (80.6–89.3)	82.0 (78.4 – 86.1)	84.4 (80.1 – 88.6)	< 0.01
**Age, n (%)**						
75–79	76,233 (29.6)	30,169 (36.9)	11,343 (22.1)	13,716 (36.1)	21,005 (24.3)	< 0.01
80–84	78,766 (30.6)	26,181 (32.0)	14,550 (28.4)	12,509 (33.0)	25,526 (29.5)	
85–89	63,894 (24.8)	16,824 (20.6)	14,359 (28.0)	8,098 (21.3)	24,613 (28.4)	
90 +	38,642 (15.0)	8,614 (10.5)	11,027 (21.5)	3,626 (9.6)	15,375 (17.8)	
**Aboriginal and Torres Strait Islander status, n (%)**						
Non-Aboriginal	256,100 (99.4)	81,332 (99.4)	51,074 (99.6)	37,697 (99.3)	85,997 (99.4)	< 0.01
Aboriginal	1435 (0.6)	456 (0.6)	205 (0.4)	252 (0.7)	522 (0.6)	
**Socioeconomic status quartiles, n(%)**						
Most disadvantaged	66,279 (25.7)	21,889 (26.8)	12,215 (23.8)	10,597 (27.9)	21,578 (24.9)	< 0.01
2	54,192 (21)	17,996 (22.0)	10,491 (20.5)	8,137 (21.4)	17,568 (20.3)	
3	46,441 (18)	14,943 (18.3)	9478 (18.5)	6,694 (17.6)	15,326 (17.7)	
4	47,350 (18.4)	14,512 (17.7)	9868 (19.2)	6,600 (17.4)	16,370 (18.9)	
Most advantaged	40,911 (15.9)	11,904 (14.6)	8687 (16.9)	5,681 (15.0)	14,639 (16.9)	
Missing	2362 (0.9)	544 (0.7)	540 (1.1)	240 (0.6)	1,038 (1.2)	
**Admission type, n (%)**						
Medical	224,949 (87.3)	71,714 (87.7)	44,897 (87.6)	32,486 (85.6)	75,852 (87.7)	< 0.01
Surgical	23,339 (9.1)	7020 (8.6)	5374 (10.5)	3,010 (7.9)	7,935 (9.2)	
Other	9247 (3.6)	3054 (3.7)	1008 (2.0)	2,453 (6.5)	2,732 (3.2)	
**Number of prior admissions over 2 years, excluding index admission, n (%)**						
0	84,775 (32.9)	43,072 (52.7)	17,527 (34.2)	11,471 (30.2)	12,705 (14.7)	< 0.01
1	62,827 (24.4)	20,433 (25.0)	14,370 (28.0)	10,092 (26.6)	17,932 (20.7)	
2 or more	109,933 (42.7)	18,283 (22.4)	19,382 (37.8)	16,386 (43.2)	55,882 (64.6)	
Median HFRS (IQR)	5.5 (1.9–12.0)	1.6 (0–3)	9.0 (6.6–13.2)	2.0 (0.7–3.4)	13.3 (8.6–20.3)	< 0.01
Median number of chronic conditions (IQR)	1 (0–3)	0 (0–1)	1 (0–1)	2 (2–3)	3 (2–5)	< 0.01

*HFRS * Hospital frailty risk score, *IQR* interquartile range.

### Frailty and multimorbidity

The HFRS ranged from 0 to 88, with 53.5% of the patients having an elevated frailty risk score, including 35.6% at intermediate and 17.9% at high risk (Supplementary Table S1). Multimorbidity was present in 124,468 (48.4%) of the study cohort, with median number of chronic conditions among multimorbid patients being 3 (interquartile range 2–4).

In our study cohort, 33.6% patients had both multimorbidity and elevated frailty risk, 19.9% had elevated frailty risk only, 14.7% had multimorbidity only and the remaining 31.8% had neither. Hospitalised patients experiencing both states tended to be older (median age 84 years), have more hospital stays (65% had two or more stays), and have both higher HFRS scores and a larger number of chronic conditions. Patients with neither factor were younger and with fewer prior admissions (Table 1).

### Outcomes

Overall 30-day mortality and readmission rates in our study were both 11%, with 30% of patients staying longer than 10 days in hospital. Crude incidence rates for each outcome were higher in those with elevated frailty risk or multimorbidity, with highest rates observed in patients who were both multimorbid and at elevated risk of frailty (Table 2).

**Table 2:** Crude patient outcomes by multimorbidity and frailty risk.

	Total	Multimorbidity by Frailty risk				
	N (%)	Neither n = 81,788	Elevated frailty risk only n = 51,279	Multimorbid only n = 37,949	Both n = 86,519	*p*-value*
Mortality within 30-days	28,886 (11.2)	3854 (4.7)	4731 (9.2)	4046 (10.7)	16,255 (18.8)	< 0.001
Median LOS (days) (IQR)	5 (2–12)	2 (1–6)	6 (2–16)	4 (2–8)	8 (3–19)	< 0.001
Prolonged LOS (> 10 days)	76,585 (29.7)	11,855 (14.5)	19,181 (37.4)	8001 (21.1)	37,548 (43.4)	< 0.001
Readmission within 30-days	26,264 (11.2)	5457 (6.9)	4727 (10.0)	3960 (11.4)	12,120 (16.5)	< 0.001

* Differences in proportions tested using χ^2^ test, and medians using Kruskal Wallis test.

**Figure 1:**
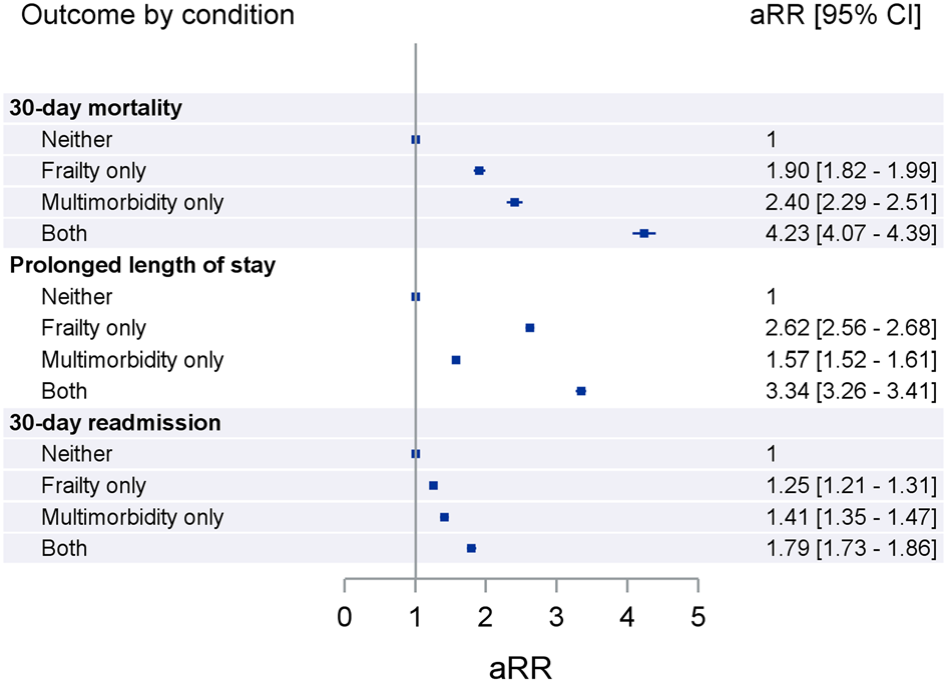
Adjusted relative risk (aRR) between multimorbidity and elevated frailty risk with adverse outcomes.

Figure 1 shows the results from the Poisson random intercept models, adjusting for age, sex, socioeconomic status and the number of prior admissions. The relative risk of adverse outcomes continued to be higher in multimorbid and elevated frailty risk individuals after risk-adjustment. Patients who experienced both health states had the highest risks of adverse outcomes. Multimorbid individuals with elevated frailty risk had 4.2 (95% CI 4.1–4.4) times higher risk of mortality, 3.3 (95% CI 3.3–3.4) times higher risk of prolonged hospital stays and 1.8 (95% CI 1.7–1.9) times higher risk of 30-day unplanned readmission than those with neither, with risks also being higher in patients with only one health state.

Interaction effect results are shown in Table 3. Significant interactions between multimorbidity and frailty were observed on both additive and multiplicative scales for mortality (RERI_RR_ 0.93 (95% CI 0.81–1.04), ratio of RR 0.93 (95% CI 0.88–0.98)) and prolonged length of stay (RERI_RR_ 0.15 (95% CI 0.09–0.21), ratio of RR 0.81 (0.79–0.84)), and additive scale only for readmission (RERI_RR_ 0.13 (95% CI 0.06–0.20), ratio of RR 1.02 (95% CI 0.96–1.07)).

**Table 3:** Additive and multiplicative Interaction effects of multimorbidity and frailty risk on adverse patient outcomes, full cohort.

Mortality within 30-days post admission	Low frailty risk			Elevated frailty risk		
	N with outcome	% outcome	aRR (95% CI)	N with outcome	%outcome	aRR (95% CI)
No multimorbidity	3854	4.7	1	4731	9.2	1.90 (1.82–1.99)
Multimorbidity	4046	10.7	2.40 (2.29–2.51)	16,255	18.8	4.23 (4.07–4.39)
^a^Measure of effect modification on additive scale: RERI (95% CI) = 0.93 (0.81–1.04)*						
^a^Measure of effect modification on multiplicative scale: ratio of RR = 0.93 (0.88–0.98), *p*-value = 0.006*						

Prolonged LOS	Low frailty risk			Elevated frailty risk		
	N with outcome	% outcome	aRR (95% CI)	N with	%outcome	aRR (95% CI)
No multimorbidity	11,855	14.5	1	19,181	37.4	2.62 (2.56–2.68)
Multimorbidity	8001	21.1	1.57 (1.52–1.61)	37,548	43.4	3.34 (3.26–3.41)
^a^Measure of effect modification on additive scale: RERI (95% CI) = 0.15 (0.09–0.21)*						
^a^Measure of effect modification on multiplicative scale: ratio of RR = 0.81 (0.79–0.84), *p*-value < 0.001*						

Readmission within 30-days post discharge	Low frailty risk			Elevated frailty risk		
	N with outcome	% outcome	aRR (95% CI)	N with outcome	%outcome	aRR (95% CI)
No multimorbidity	5457	6.9	1	4727	9.9	1.25 (1.21–1.31)
Multimorbidity	3960	11.4	1.41 (1.35–1.47)	12,120	16.5	1.79 (1.73–1.86)
^a^Measure of effect modification on additive scale: RERI (95% CI) = 0.13 (0.06–0.20)*						
^a^Measure of effect modification on multiplicative scale: ratio of RR = 1.02 (0.96–1.07), *p*-value = 0.57						

*Denotes significance at 5% level.

^a^Significance of an interaction on an additive scale is denoted where RERI is different from 0, and on the multiplicative scale if ratio of RR is different from 1.

### Stratified analysis

We found similar interaction effects for 30-day mortality and readmissions when stratified by admission type, although with larger effect sizes for surgical patients, among whom those with both multimorbidity and elevated frailty had a high risk of 30-day mortality (aRR: 7.2; 95% CI 6.1–8.5) and 30-day readmission (aRR: 2.6; 95% CI 2.3–3.0) compared with those with neither (Supplementary Table S2). The results for prolonged length of stay in this group were attenuated in the surgical cohort compared with the medical cohort (Supplementary Tables S3).

## Discussion

Our study shows that multimorbidity and frailty risk occur both jointly and in isolation in our cohort of older Australians with unplanned admissions to hospital, and have varying and interacting effects on adverse health outcomes. We observed the largest adverse effects on mortality, readmission and prolonged lengths of stay in those patients with both multimorbidity and elevated frailty risk, with greater risks of mortality and readmission in surgical patients. To our knowledge, no prior studies have explored the nature of any interactions between HFRS and multimorbidity in hospital patients, nor reported their joint effects. Our findings highlight that when identifying older hospitalised patients at risk of complications, accounting for both the patient’s burden of chronic disease and vulnerability to stressors of treatment will both increase the breadth of patients identified, and help separate those at even higher risk of complication.

Measures of additive interaction are not frequently reported in the literature, despite their value for identifying individuals who would most benefit from treatment or need more monitoring for complications or adverse health outcomes^17^. Our findings, congruent with prior research^7,20–23^, show that both multimorbidity and frailty have important independent impacts on health outcomes, but further demonstrate that their joint effects on mortality and length of stay are amplified, while their effects on readmission are additive. The different scale of interaction between outcomes may reflect the fact that readmissions are only measured for patients who survived to discharge (in our study 91% of patients) – thus attenuating some of the excess risk. Our findings indicate that the presence of not only multimorbidity and frailty, but also their co-occurrence, in patient populations are important inputs to hospital care resource projections and planning.

Our study also highlights that, although a high proportion of elderly patients experience both health states, a notable portion of hospitalised patients are multimorbid without exhibiting elevated frailty risk, and vice versa. Multimorbid-only individuals had higher age-adjusted mortality and readmission risks than frail-only individuals, reflecting the ongoing risk of complications experienced by people with multiple chronic diseases. Conversely, patients with elevated frailty risk had higher risk of prolonged hospitalisation than multimorbid-only patients, reflecting the vulnerability of these patients to acute complications of care. Using only one of these factors to identify at-risk patients will not only fail to account for the interactive effect found in this study, but also potentially fail to identify patients at-risk of different types of complications.

A recent systematic review by Vetrano et al.^14^ reported that 72% of frail individuals had multimorbidity, and 16% of multimorbid individuals were frail. In our study these proportions were 63% and 70% respectively. The differences in our estimates could be attributed to the study populations and methods of frailty assessment. We studied older hospitalised patients presenting as unplanned cases, with frailty risk ascertained from routinely collected data. The majority of the studies included in the systematic review investigated community dwelling participants, with frailty ascertained using Fried et al. Frail Phenotype^24^ based on physical signs and symptoms.

There are several limitations to our study. First, while the indices of multimorbidity and frailty are commonly used and validated, there remain aspects of patient frailty that are unable to be captured in administrative data, as well as possible under-ascertainment of some morbidities. Second, there is overlap between the ICD-10 diagnoses codes used in frailty risk and comorbidity ascertainment which makes it difficult to disentangle their effects, although prior research indicates only a weak correlation between HFRS and the Charlson Index^25^. Third, there might be under-ascertainment of frailty and multimorbidity in patients with few or no prior hospitalisations. Lastly, our restriction to unplanned admissions limits generalisability of our findings to broader patient populations such as those undergoing elective surgery.

The strengths of this study include its large size and the use of linked population-based data on hospitalisations and mortality, enabling calculation of patient outcomes within and beyond the index hospital stay. Furthermore, ascertainment of multimorbidity and HFRS used administrative data only and ICD-10 diagnosis codes, presenting an approach that is amenable to incorporation into hospital forecasting planning and models.

## Conclusion

Multimorbidity and frailty coexist in older hospitalised patients and interact to increase the risk of adverse outcomes beyond the sum of their individual effects. The risk of mortality, readmission and prolonged lengths of stay among multimorbid individuals with elevated frailty risk is two to four times higher than for those without either factor, and larger than in patients with only one factor. Joint effects of multimorbidity and frailty should be considered in health outcomes research and when administering hospital resources.

## Methods

### Study design

We conducted a retrospective cohort study using routinely collected administrative hospital and mortality data.

### Setting and data

New South Wales (NSW) is Australia’s most populous state with 7.2 million residents in 2012^26^. We used NSW Admitted Patient Data collection (hospital data) linked with mortality data for a period 1 January 2008 – 31 March 2013. The hospital data included records for all public and private hospital admissions ending in discharge, transfer, type change or death. Hospital admissions were coded using the International Statistical Classification of Diseases and Related Problems, tenth revision, Australian modification (ICD-10-AM) and Australian Refined Diagnosis Related Group (AR-DRG) codes^27^. The Centre for Health Record Linkage linked the two datasets using probabilistic methods, with a false positive and false negative rates of 0.5%^28^.

### Study cohort construction

Our study replicated inclusion criteria of the original HFRS publication^9^ with a cohort containing NSW residents, aged 75 and over, having at least one unplanned admission to an acute hospital during 1 January 2010 – 31 December 2012. For admissions ending in type change (e.g. from acute to sub-acute care) or transfer, contiguous periods of stay were constructed using admission dates and admission status from the first episode and separation dates and separation type from the last episode of care. We selected a single random hospital stay for each patient as their ‘index’ admission.

### Predictors and outcomes

We classified the two main analysis variables of interest, multimorbidity and frailty risk, using the ICD-10-AM diagnoses codes from the index admission and any hospitalisations in the preceding two-year period.

Long term conditions were ascertained from a list of 29 chronic conditions from the Charlson and Elixhauser indices^29^, supplemented with core morbidities from more recent systematic reviews^30^^31^ (Supplementary Table S4). Multimorbidity was defined as having at least two conditions from this list.

We calculated a continuous HFRS using the 109 ICD-10 codes from Gilbert et al.^9^, adapted to the Australian modification (ICD-10-AM) (Supplementary Table S5). The HFRS captures comorbidities associated with frailty, as well as functional deficits and symptoms. Presence of each of the 109 ICD-10 codes was ascertained from patients’ hospital records, assigned a weight, and weights were summed across all the codes to yield the HFRS^9^. We created dichotomous frailty groups of low frailty (HFRS < 5) and elevated frailty risk (HFRS ≥ 5, combining intermediate and high frailty) using the validated cut points from Gilbert et al.^9^.

We constructed a composite variable of multimorbidity and frailty risk with four categories: neither multimorbid nor at elevated risk of frailty, elevated frailty risk only, multimorbid only, and both multimorbid and with elevated frailty risk.

Other covariates included age at index admission (in five-year age groups), sex, quantiles of socioeconomic status based on the Australian Bureau of Statistics Socioeconomic Indices of Areas (SEIFA) Index of Relative Socio-economic Advantage and Disadvantage (IRSAD), and number of hospital admissions in the preceding two-year period (none, one, two or more).

Outcomes of interest included: mortality within 30 days of index admission; prolonged hospital stay (> 10 days in hospital); unplanned readmission within 30 days of discharge (for patients discharged alive), in line with the original HFRS development study^9^. Results were stratified by admission type, grouped into medical (not involving an operating room procedure), surgical (involving significant operating room procedure) and other (involving non-operating room procedure) admissions based on AR-DRG procedures^32^.

### Statistical analysis

We used descriptive statistics to compare demographic characteristics and crude outcome proportions between multimorbidity and frailty risk groups.

We built Poisson random intercept models to quantify the association of outcomes with multimorbidity and frailty accounting for clustering within hospitals, and adjusted for age, sex, socio-economic status, and number of prior admissions. Effects are reported as relative risks (RR), given the high frequency of the outcomes^33,34^.

We calculated and presented the analyses of interaction as recommended by Knol and VanderWeele^16^. Interaction on an additive scale was estimated using the relative excess risk due to interaction (RERI_RR_), with adjustments for clustered data^35^. RERI_RR_ = 0 implies no interaction (exact additivity), RERI_RR_ > 0 denotes interaction more than additivity and RERI_RR_ < 0 means interaction less than additivity. Interaction on a multiplicative scale was assessed via the inclusion of an interaction term in the adjusted Poisson model including both main effects (multimorbidity and frailty) and interaction term (multimorbidity*frailty). Significance of an interaction on the multiplicative scale is denoted where relative risk of the interaction term is different from 1, and on additive scale if RERI is different from 0.

We used SAS version 9.4 (SAS Institute Inc., Cary, NC) for data management, analysis and graph creation.

### Ethical approval

We obtained ethics approvals from the NSW Population and Health Services Research (reference 2009/03/141) and the Aboriginal Health and Medical Research Council (reference 684/09) Ethics Committees, with a waiver for written informed consent. The study was conducted in accordance with the Australian National Health and Medical Research Council’s National Statement on Ethical Conduct in Human Research^36^.

### Data availability

The data sets used in this article are available from the NSW Ministry of Health and the Register of Births, Deaths and Marriages, NSW, Australia. The data sets were constructed with the permission of each of the source data custodians and with specific ethical approvals. The authors do not have permission to share individual unit record data due to its highly confidential nature. The data are available to researchers on request, and subject to approval processes from data custodians and ethics committees, as outlined on the NSW Centre for Health Record linkage website (https://www.cherel.org.au/apply-for-linked-data).

## Supplementary Information

Supplementary Information.
