# Supplementary material for: Interaction effects of multimorbidity and frailty on adverse health outcomes in elderly hospitalised patients
Source: Sci Rep. 2022 Aug 19;12:14139. doi: 10.1038/s41598-022-18346-x (PMC9391344; doi:10.1038/s41598-022-18346-x)
Supplement: Supplementary file 1 — Supplementary Information. [file 41598_2022_18346_MOESM1_ESM.pdf]

Supplementary Tables

Table S1. Baseline characteristics by multimorbidity and frailty at index hospital stay (2010 – 2012)

Table S2. Additive and multiplicative Interaction effects of multimorbidity and frailty risk on adverse patient outcomes, surgical cohort

Table S3. Additive and multiplicative Interaction effects of multimorbidity and frailty risk on adverse patient outcomes, medical cohort

Table S4. List of ICD-10 codes for morbidity ascertainment

Table S5. List of ICD-10 codes for HFRS calculation

Supplementary Table S1. Baseline characteristics by multimorbidity and frailty at index hospital stay (2010 – 2012)

	Total	Frailty risk category			Multimorbidity		
		Low risk	*Elevated risk*	*p-value*	*No*	*Yes*	*p-value*
	N = 257,535	***n = 119,737***	***n = 137,798***		***n = 133,067***	***n = 124,468***	
**Sex, n (%)**							
Male	110,125 (42.8)	53,950 (45.1)	56,175 (40.8)	<0.01	51,497 (38.7)	58,628 (47.1)	<0.01
Female	147,410 (57.2)	65,787 (54.9)	81,623 (59.2)		81,570 (61.3)	65,840 (52.9)	
**Median age [IQR]**							
	83.3 [79.2 – 87.7]	81.9 [78.3 – 86.2]	84.6 [80.3 – 88.8]	<0.01	83.0 [79.0 – 87.5]	83.6 [79.5 – 87.9]	<0.01
**Age, n (%)**							
75-79	76,233 (29.6)	43,885 (36.7)	32,348 (23.5)	<0.01	41,512 (31.2)	34,721 (27.9)	<0.01
80-84	78,766 (30.6)	38,690 (32.3)	40,076 (29.1)		40,731 (30.6)	38,035 (30.6)	
85-89	63,894 (24.8)	24,922 (20.8)	38,972 (28.3)		31,183 (23.4)	32,711 (26.3)	
90+	38,642 (15)	12,240 (10.2)	26,402 (19.2)		19,641 (14.8)	19,001 (15.3)	
**Aboriginal and Torres Strait Islander status, n (%)**							
Non-Aboriginal	256,100 (99.4)	119,029 (99.4)	137,071 (99.5)	0.03	132,406 (99.5)	123,694 (99.4)	<0.01
Aboriginal	1,435 (0.6)	708 (0.6)	727 (0.5)		661 (0.5)	774 (0.6)	
**Socioeconomic status quartiles, n(%)**							
Most disadvantaged	66,279 (25.7)	32,486 (27.1)	33,793 (24.5)	<0.01	34,104 (25.6)	32,175 (25.9)	<0.01
2	54,192 (21)	26,133 (21.8)	28,059 (20.4)		28,487 (21.4)	25,705 (20.7)	
3	46,441 (18)	21,637 (18.1)	24,804 (18)		24,421 (18.4)	22,020 (17.7)	
4	47,350 (18.4)	21,112 (17.6)	26,238 (19)		24,380 (18.3)	22,970 (18.5)	
Most advantaged	40,911 (15.9)	17,585 (14.7)	23,326 (16.9)		20,591 (15.5)	20,320 (16.3)	
Missing	2,362 (0.9)	784 (0.7)	1,578 (1.1)		1,084 (0.8)	1,278 (1)	
**Admission type, n (%)**							
Medical	224,949 (87.3)	104,200 (87)	120,749 (87.6)	<0.01	116,611 (87.6)	108,338 (87)	<0.01
Surgical	23,339 (9.1)	10,030 (8.4)	13,309 (9.7)		12,394 (9.3)	10,945 (8.8)	
Other	9,247 (3.6)	5,507 (4.6)	3,740 (2.7)		4,062 (3.1)	5,185 (4.2)	

**Number of prior admissions over 2 years, excluding index admission, n (%)**							
0	84,775 (32.9)	54,543 (45.6)	30,232 (21.9)	<0.01	60,599 (45.5)	24,176 (19.4)	<0.01
1	62,827 (24.4)	30,525 (25.5)	32,302 (23.4)		34,803 (26.2)	28,024 (22.5)	
2 or more	109,933 (42.7)	34,669 (29)	75,264 (54.6)		37,665 (28.3)	72,268 (58.1)	
**HFRS category**							
Low risk	119,737 (46.5)	119,737 (100)			81,788 (61.5)	37,949 (30.5)	<0.01
Elevated risk	137,798 (53.5)		137,798 (100)		51,279 (38.5)	86,519 (69.5)	
**Multimorbidity**							
No	133,067 (51.7)	81,788 (68.3)	51,279 (37.2)	<0.01	133,067 (100)		
Yes	124,468 (48.3)	37,949 (31.7)	86,519 (62.8)			124,486 (100)	
**Median HFRS [IQR]**							
	5.5<br>[1.9 – 12.0]	1.8<br>[0 – 3.2]	11.3<br>[7.6 – 17.6]	<0.01	3.4<br>[1.1 – 7.5]	9.1<br>[3.9 – 16.7]	<0.01
**Median number of chronic conditions [IQR]**							
	1<br>[0 – 3]	1<br>[0 – 2]	2<br>[1 – 4]	<0.01	0<br>[0 – 1]	3<br>[2 – 4]	<0.01

HFRS – Hospital frailty risk score, IQR – interquartile range

Supplementary Table S2. Additive and multiplicative Interaction effects of multimorbidity and frailty risk on adverse patient outcomes, surgical cohort

**Mortality within 30-days post discharge**	**Non-frail**			**Frail**		
	N with outcome	% outcome	aRR (95% CI)	N with outcome	% outcome	aRR (95% CI)
**No multimorbidity**	171	2.4	1	379	7.1	2.75 (2.29 – 3.31)
**Multimorbidity**	229	7.6	3.21 (2.63 – 3.93)	1,339	16.9	7.24 (6.14 – 8.54)

*Measure of effect modification on additive scale: RERI (95% CI) = 2.27 (1.58 – 2.96) **

Measure of effect modification on multiplicative scale: ratio of RR= 0.82 (0.65 – 1.03), p-value =0.086

**Prolonged LOS**	**Non-frail**			**Frail**		
	N with outcome	% outcome	aRR (95% CI)	N with outcome	% outcome	aRR (95% CI)
**No multimorbidity**	2,735	39.0	1	3,921	73.0	1.76 (1.67 – 1.85)
**Multimorbidity**	1,239	41.2	1.05 (0.98 – 1.12)	6,088	76.7	1.91 (1.82 – 2.00)

*Measure of effect modification on additive scale: RERI (95% CI) =0.10 (0.00 – 0.20)**

Measure of effect modification on multiplicative scale: ratio of RR=1.03 (0.96 – 1.12) , p-value =0.391

**Readmission within 30-days post discharge**	**Non-frail**			**Frail**		
	N with outcome	% outcome	aRR (95% CI)	N with outcome	% outcome	aRR (95% CI)
**No multimorbidity**	281	4.1	1	356	7.0	1.64 (1.40 – 1.92)
**Multimorbidity**	263	9.4	1.99 (1.68 – 2.36)	863	13.2	2.63 (2.28 – 3.03)

Measure of effect modification on additive scale: RERI (95% CI) =0.01 (-0.36 – 0.37)

*Measure of effect modification on multiplicative scale: ratio of RR= 0.81 (0.66 – 1.00), p-value =0.046**

* Significant at 5% level. [a] Significance of an interaction on an additive scale is denoted where RERI is different from 0, and on the multiplicative scale if ratio of RR is different from 1.

Supplementary Table S3. Additive and multiplicative Interaction effects of multimorbidity and frailty risk on adverse patient outcomes, medical cohort

Mortality within 30-days post discharge	Non-frail			Frail		
	N with outcome	% outcome	aRR (95% CI)	N with outcome	% outcome	aRR (95% CI)
**No multimorbidity**	3,572	5.0	1	4,227	9.4	1.84 (1.76 – 2.92)
**Multimorbidity**	3,596	11.1	2.35 (2.25 – 2.47)	14,267	18.8	4.00 (3.84 – 4.16)

*Measure of effect modification on additive scale: RERI (95% CI) = 0.81 (0.69 – 0.93)**

*Measure of effect modification on multiplicative scale: ratio of RR= 0.92 (0.87 – 0.98), p-value =0.008**

Prolonged LOS	Non-frail			Frail		
	N with outcome	% outcome	aRR (95% CI)	N with outcome	% outcome	aRR (95% CI)
**No multimorbidity**	6,726	9.4	1	11,819	26.3	2.80 (2.73 – 2.88)
**Multimorbidity**	4,642	14.3	1.68 (1.62 – 1.73)	24,096	31.8	3.70 (3.61 – 3.80)

*Measure of effect modification on additive scale: RERI (95% CI) =0.23 (0.15 – 0.30)**

*Measure of effect modification on multiplicative scale: ratio of RR= 0.79 (0.76 – 0.82), p-value <0.001**

Readmission within 30-days post discharge	Non-frail			Frail		
	N with outcome	% outcome	aRR (95% CI)	N with outcome	% outcome	aRR (95% CI)
**No multimorbidity**	4,996	7.2	1	4,272	10.2	1.23 (1.18 – 1.28)
**Multimorbidity**	3,461	11.7	1.36 (1.31 – 1.43)	10,835	16.8	1.72 (1.66 – 1.79)

*Measure of effect modification on additive scale: RERI (95% CI) =0.13 (0.06 – 0.20)**

Measure of effect modification on multiplicative scale: ratio of RR=1.03 (0.97 – 1.09), p-value =0.367

* Significant at 5% level. Significance of an interaction on an additive scale is denoted where RERI is different from 0, and on the multiplicative scale if ratio of RR is different from 1.

Supplementary Table S4: List of ICD-10 codes for multimorbidity ascertainment, and prevalence within cohort and by multimorbidity and frailty risk status

Name	ICD-10-AM Codes	Source	Cohort		% within group			
			N	%	Neither	Frail only	MM only	Both
**AIDS/HIV**	B20.x-B22.x, B24.x	Elixhauser	23	0.0	<5	<5	26	70
**Alcohol abuse***	F10.x*, E52.x, G62.1, I42.6, K29.2, K70.0, K70.3, K70.9, T51.x, Z50.2*, Z71.4, Z72.1	Elixhauser	4,028	1.6	8	11	13	68
**Asthma and bronchiectasis**	J45.x, J46.x, J47.x	New	5,063	2.0	14	5	27	54
**Cancer**	C00.x -C26.x, C30.x -C34.x, C37.x -C41.x, C43.x, C45.x -C58.x, C60.x -C76.x, C77.x -C80.x, C81.x-C85.x, C88.x, C96.x, C90.0, C90.2, C97.x	Elixhauser (combining: ‘Lymphoma’, ‘Metastatic cancer’ and ‘Solid tumor without metastasis’)	24,036	9.3	18	13	18	51
**Cardiac arrhythmias, including atrial fibrillation***	I44.1-I44.3, I45.6, I45.9, I47.x-I49.x, R00.0*, R00.1*, R00.8*, T82.1, Z45.0, Z95.0	Elixhauser Index	69,883	27.1	11	6	24	59
**Cerebrovascular disease, including stroke/TIA (CVD)***	G45.x*, G46.x, H34.0, I60.x-I69.x*	Charlson Index	27,497	10.7	7	4	16	74
**Chronic IHD**	I25.x	New	26,050	10.1	4	1	40	56
**Chronic kidney disease***	N00.x–N08.x, N11.x, N12.x, N14.x–N16.x, N18.x*, N19.x*, N25.x–N28.x*, N39.1*, N39.2*, Q60.x–Q63.x, T82.4, T86.1, V56.0, V56.8, V42.0, V45.1, Z49.x, Z94.0, Z99.2*	New –expanded version of renal disease in both Charlson and Elixhauser indices	29,169	11.3	2	5	13	80

**Chronic pulmonary disease**	I27.8, I27.9, J40.x -J44.x, J60.x -J67.x, J68.4, J70.1, J70.3	Elixhauser (excluding J45-J47, now in a new category: 'Asthma or bronchiectasis')	24,227	9.4	14	6	25	56
**Coagulopathy**	D65.x-D68.x, D69.1, D69.3-D69.6	Elixhauser	11,079	4.3	5	4	17	74
**Congestive heart failure**	I09.9, I11.0, I13.0, I13.2, I42.0, I42.5-I42.9, I43.x, I50.x, P29.0	Elixhauser (excluding I25.5, included in the new category CIHD above)	37,770	14.7	4	3	26	68
**Dementia***	F00.x-F03.x*, F05.1*, G30.X*, G31.1*	Charlson	33,319	12.9	4	25	5	66
**Depression***	F20.4, F31.3-F31.5, F32.x*, F33.x, F34.1, F41.2, F43.2	Elixhauser	7,585	2.9	6	7	11	77
**Diabetes**	E10.x- E14.x	Elixhauser (combining uncomplicated and complicated diabetes)	32,374	12.6	8	5	25	63
**Drug abuse**	F11.x -F16.x, F18.x, F19.x, Z71.5, Z72.2	Elixhauser	603	0.2	2	7	9	81
**Epilepsy***	G40.x*, G41.x	New	1,523	0.6	5	9	9	77
**Hypertension**	I10.x -I13.x, I15.x	Elixhauser (combining uncomplicated and complicated hypertension)	77,940	30.3	6	4	27	63
**Hypothyroidism**	E00.x -E03.x, E89.0	Elixhauser	3,007	1.2	3	5	17	75
**Liver disease**	B18.x, I85.x, I86.4, I98.2, K70.x, K71.1, K71.3-K71.5, K71.7, K72.x -K74.x, K76.0, K76.2-K76.9, Z94.4	Elixhauser	2,595	1.0	5	4	19	71
**Multiple sclerosis**	G35.x, G36.x, G367.x, H46.x	New	56	0.0	11	9	18	63

**Myocardial infarction**	I21.x, I22.x	Charlson (excluding I25.2 included in the new category CIHD above)	19,227	7.5	3	1	35	61
**Parkinson's***	G20.x*, G21.x, G22.x	New	5,203	2.0	4	14	7	75
**Peptic ulcer disease excluding bleeding***	K25.7, K25.9, K26.7*, K26.9*, K27.7, K27.9, K28.7, K28.9	Elixhauser Index	1,967	0.8	13	8	18	61
**Peripheral vascular disorders**	I70.x, I71.x, I73.1, I73.8, I73.9, I77.1, I79.0, I79.2, K55.1, K55.8, K55.9, Z95.8, Z95.9	Elixhauser Index	11,724	4.6	8	4	22	65
**Psychoses**	F20.x, F22.x -F25.x, F28.x, F29.x, F30.2, F31.2, F31.5	Elixhauser Index	1,493	0.6	8	7	13	71
**Pulmonary circulation disorders**	I26.x, I27.x, I28.0, I28.8, I28.9	Elixhauser Index	9,290	3.6	6	3	25	67
**Rheumatoid arthritis / collagen vascular diseases**	L94.0, L94.1, L94.3, M05.x, M06.x, M08.x, M12.0, M12.3, M30.x, M31.0-M31.3, M32.x -M35.x, M45.x, M46.1, M46.8, M46.9	Elixhauser Index	3,253	1.3	8	10	16	66
**Valvular disease**	A52.0, I05.x -I08.x, I09.1, I09.8, I34.x -I39.x, Q23.0-Q23.3, Z95.2-Z95.4	Elixhauser Index	11,635	4.5	3	2	29	67
**Paralysis***	G04.1, G11.4, G80.1, G80.2, G81.x*, G82.x, G83.0–G83.4, G83.9	Elixhauser Index	12,086	4.7	0	1	3	95

* Overlapping ICD-10 codes with HFRS diagnoses

Supplementary Table S5: List of ICD-10 codes for HFRS calculation, and prevalence within cohort and by multimorbidity and frailty risk status

ICD-10 code	Description	Cohort		% within group			
		N	%	Neither	Frail only	MM only	Both
**A04**	Other bacterial intestinal infections	2348	0.9	10	24	3	63
**A09**	Diarrhoea and gastroenteritis of presumed infectious origin	17,792	6.9	16	24	5	54
**A41**	Other septicaemia	13,205	5.1	5	22	3	69
**B95**	Streptococcus and staphylococcus as the cause of diseases classified to other chapters	17,635	6.8	6	24	3	68
**B96**	Other bacterial agents as the cause of diseases classified to other chapters (secondary code)	39,039	15.2	2	34	1	63
**D64**	Other anaemias	24,300	9.4	11	20	9	61
**E05**	Thyrotoxicosis [hyperthyroidism]	1,331	0.5	8	16	13	63
**E16**	Other disorders of pancreatic internal secretion	971	0.4	7	19	4	69
**E53**	Deficiency of other B group vitamins	2,778	1.1	4	30	3	63
**E55**	Vitamin D deficiency	4,801	1.9	4	29	3	64
**E83**	Disorders of mineral metabolism	10,255	4.0	7	19	7	67
**E86**	Volume depletion	39,117	15.2	8	28	3	62
**E87**	Other disorders of fluid, electrolyte and acid- base balance	40,037	15.5	6	23	5	67
**F00***	Dementia in Alzheimer's disease	6,408	2.5	0	35	0	65
**F01***	Vascular dementia	2,995	1.2	3	17	4	76
**F03***	Unspecified dementia	22,837	8.9	5	24	5	66
F05*	Delirium, not induced by alcohol and other psychoactive substances	21,983	8.5	2	29	1	69
F10*	Mental and behavioural disorders due to use of alcohol	3,417	1.3	8	12	11	68
F32*	Depressive episode	5,986	2.3	5	7	9	78
G20*	Parkinson's disease	4,986	1.9	4	14	7	75
G30*	Alzheimer's disease	6,460	2.5	0	34	0	66
G31*	Other degenerative diseases of nervous system, not elsewhere classified	1,680	0.7	6	19	4	71
G40*	Epilepsy	1,423	0.6	5	9	9	77
G45*	Transient cerebral ischaemic attacks and related syndromes	7,682	3.0	15	6	21	58
G81*	Hemiplegia	11,292	4.4	0	1	2	97
H54	Blindness and low vision	3,197	1.2	6	24	5	66
H91	Other hearing loss	3,198	1.2	6	25	5	64
I63*	Cerebral Infarction	8,471	3.3	2	2	11	84
I67*	Other cerebrovascular diseases	1,643	0.6	2	5	6	86
I69*	Sequelae of cerebrovascular disease (secondary codes)	4,506	1.7	0	2	1	97
I95	Hypotension	38,267	14.9	8	22	7	63
J18	Pneumonia, organism unspecified	28,718	11.2	16	17	11	56
J22	Unspecified acute lower respiratory infection	15,497	6.0	15	17	11	57
J69	Pneumonitis due to solids and liquids	7,831	3.0	6	19	3	72
J96	Respiratory failure, not elsewhere classified	7,695	3.0	7	12	9	71
K26*	Duodenal ulcer	1,520	0.6	15	18	8	59

K52	Other noninfective gastroenteritis and colitis	4,109	1.6	15	20	8	57
K59	Other functional intestinal disorders	33,072	12.8	10	28	4	58
K92	Other diseases of digestive system	14,405	5.6	23	17	10	50
L03	Cellulitis	19,021	7.4	15	23	5	57
L08	Other local infections of skin and subcutaneous tissue	1,068	0.4	11	22	5	62
L89	Decubitus ulcer	12,691	4.9	3	24	2	71
L97	Ulcer of lower limb, not elsewhere classified	8,187	3.2	5	23	3	69
M15	Polyarthrosis	597	0.2	8	22	6	63
M19	Other arthrosis	4,154	1.6	8	22	7	62
M25	Other joint disorders, not elsewhere classified	9,564	3.7	12	29	4	55
M41	Scoliosis	607	0.2	11	30	3	57
M48	Spinal stenosis (secondary code only)	4,155	1.6	17	27	7	49
M79	Other soft tissue disorders, not elsewhere classified	5,069	2.0	15	22	6	57
M80	Osteoporosis with pathological fracture	21,568	8.4	20	20	12	47
M81	Osteoporosis without pathological fracture	15,348	6.0	12	21	10	56
N17	Acute renal failure	28,079	10.9	3	17	5	76
N18*	Chronic renal failure	24,658	9.6	1	4	12	83
N19*	Unspecified renal failure	3,191	1.2	2	7	11	80
N20	Calculus of kidney and ureter	1,588	0.6	30	19	9	43
N28*	Other disorders of kidney and ureter, not elsewhere classified	1,350	0.5	5	8	13	74
N39*	Other disorders of urinary system (includes urinary tract infection and urinary incontinence)	50,226	19.5	3	36	1	61
R00*	Abnormalities of heart beat	15,834	6.1	12	8	18	62
R02	Gangrene, not elsewhere classified	643	0.2	8	20	3	68
R11	Nausea and vomiting	14,826	5.8	19	22	8	51
R13	Dysphagia	15,837	6.1	7	18	4	70
R26	Abnormalities of gait and mobility	12,644	4.9	4	28	2	66
R29	Other symptoms and signs involving the nervous and musculoskeletal systems (R29·6 Tendency to fall)	18,269	7.1	2	30	1	68
R31	Unspecified haematuria	7,939	3.1	10	25	4	61
R32	Unspecified urinary incontinence	19,010	7.4	3	26	2	69
R33	Retention of urine	15,590	6.1	9	26	4	61
R40	Somnolence, stupor and coma	3,526	1.4	4	22	2	72
R41	Other symptoms and signs involving cognitive functions and awareness	20,367	7.9	4	28	2	66
R44	Other symptoms and signs involving general sensations and perceptions	1,727	0.7	5	23	3	68
R45	Symptoms and signs involving emotional state	3,690	1.4	3	21	3	73
R47	Speech disturbances, not elsewhere classified	10,193	4.0	4	7	7	82
R50	Fever of unknown origin	6,824	2.6	16	20	10	54
R54	Senility	1,373	0.5	5	30	3	62
R55	Syncope and collapse	19,639	7.6	21	25	8	47
R56	Convulsions, not elsewhere classified	2,789	1.1	9	20	3	69
R63	Symptoms and signs concerning food and fluid intake	6,732	2.6	12	23	6	60
R69	Unknown and unspecified causes of morbidity	234	0.1	20	22	5	53
R79	Other abnormal findings of blood chemistry	1,219	0.5	16	19	9	56
R94	Abnormal results of function studies	6,972	2.7	9	20	8	63
S00	Superficial injury of head	10,134	3.9	5	42	0	53
S01	Open wound of head	11,939	4.6	14	36	2	48

S06	Intracranial injury	5,133	2.0	7	39	1	53
S09	Other and unspecified injuries of head	2,606	1.0	12	33	2	54
S22	Fracture of rib(s), sternum and thoracic spine	6,206	2.4	11	35	2	52
S32	Fracture of lumbar spine and pelvis	7,682	3.0	11	40	2	47
S42	Fracture of shoulder and upper arm	4,887	1.9	13	41	1	45
S51	Open wound of forearm	5,935	2.3	11	30	2	56
S72	Fracture of femur	15,651	6.1	14	37	2	47
S80	Superficial injury of lower leg	3,937	1.5	8	37	1	54
T83	Complications of genitourinary prosthetic devices, implants and grafts	3,098	1.2	2	27	1	70
U80	Agent resistant to penicillin and related antibiotics	0	0.0	0	0	0	0
W01	Fall on same level from slipping, tripping and stumbling	25,913	10.1	23	31	3	43
W06	Fall involving bed	4,648	1.8	9	29	2	60
W10	Fall on and from stairs and steps	4,362	1.7	27	33	3	37
W18	Other fall on same level	21,710	8.4	10	35	2	53
W19	Unspecified fall	21,559	8.4	5	38	1	57
X59	Exposure to unspecified factor	5,095	2.0	12	26	3	58
Y84	Other medical procedures as the cause of abnormal reaction of the patient	10,872	4.2	8	18	10	64
Y95	Nosocomial condition	1,156	0.4	2	18	2	77
Z22	Carrier of infectious disease	3,024	1.2	5	17	3	75
Z50*	Care involving use of rehabilitation procedures	25,433	9.9	3	23	2	72
Z60	Problems related to social environment	29,613	11.5	12	29	6	52
Z73	Problems related to life-management difficulty	789	0.3	9	27	4	61
Z74	Problems related to care-provider dependency	23,475	9.1	6	27	3	65
Z75	Problems related to medical facilities and other health care	17,564	6.8	6	22	6	66
Z87	Personal history of other diseases and conditions	4,131	1.6	29	23	13	36
Z91	Personal history of risk-factors, not elsewhere classified	4,493	1.7	8	14	10	68
Z93	Artificial opening status	3,634	1.4	9	24	4	63
Z99*	Dependence on enabling machines and devices	3,198	1.2	6	17	7	70

* Overlapping ICD-10 codes with multimorbidity list
